# Effects of the Qinghai-Tibet Railway on the Landscape Genetics of the Endangered Przewalski’s Gazelle (*Procapra przewalskii*)

**DOI:** 10.1038/s41598-017-18163-7

**Published:** 2017-12-21

**Authors:** He Yu, Shiya Song, Jiazi Liu, Sheng Li, Lu Zhang, Dajun Wang, Shu-Jin Luo

**Affiliations:** 1 Peking-Tsinghua Center for Life Sciences, Peking-Tsinghua-NIBS (PTN) Graduate Program, Peking University, Beijing, 100871 China; 2 School of Life Sciences, Peking University, Beijing, 100871 China; 3 Present Address: School of Life Sciences, Sun Yat-Sen University, Guangzhou, 510275 China

## Abstract

The Przewalski’s gazelle (*Procapra przewalskii*) is one of the most endangered ungulates in the world, with fewer than 2,000 individuals surviving in nine habitat fragments on the Qinghai-Tibet Plateau and isolated by human settlements and infrastructure. In particular, the Qinghai-Tibet railway, which crosses the largest part of the gazelle’s distribution, remains a major concern because of its potential to intensify landscape genetic differentiation. Here, using mtDNA sequencing and microsatellite genotyping to analyze 275 Przewalski’s gazelle samples collected throughout the range, we observed low level of genetic diversity (mtDNA π = 0.0033) and strong phylogeographic structure. Overall, the nine patches of gazelles can be further clustered into five populations, with a strong division between the eastern vs. western side of Qinghai Lake. Our study provides the first evidence of the genetic divergence between the Haergai North and Haergai South gazelle populations, corresponding to the recent construction of a wired enclosure along the Qinghai-Tibet railway less than ten years ago, an equivalent of five generations. Well-designed wildlife corridors across the railway along with long-term monitoring of the anthropogenic effects are therefore recommended to alleviate further habitat fragmentation and loss of genetic diversity in Przewalski’s gazelle.

## Introduction

Endemic to the Qinghai-Tibet Plateau of China, the Przewalski’s gazelle (*Procapra przewalskii* Büchner, 1891) is arguably one of the most endangered large mammals in the world. Historically, *P. przewalskii* was widespread across the semiarid grassland steppe in northeastern China but has suffered a severe population decline in the past century owing to illegal hunting, livestock competition, and habitat loss and fragmentation on an increasingly human-dominated landscape^1,2^. The first range-wide survey of the Przewalski’s gazelle, which was conducted in 1986 and repeated in the 1990s, estimated the number of free-ranging animals to be an alarmingly low 300^3,4^, and the species was denoted Critically Endangered on the IUCN Red List of Threatened Species in 1996. Three new populations that were discovered in 2003 doubled the population size to 602^4^ and the species has been downgraded to Endangered since 2008. The species now occurs as nine isolated populations around the Qinghai Lake region in northeastern Qinghai Province^5^, numbering at 1300–1600 individuals in total, according to the latest studies^6,7^.

**Figure 1:**
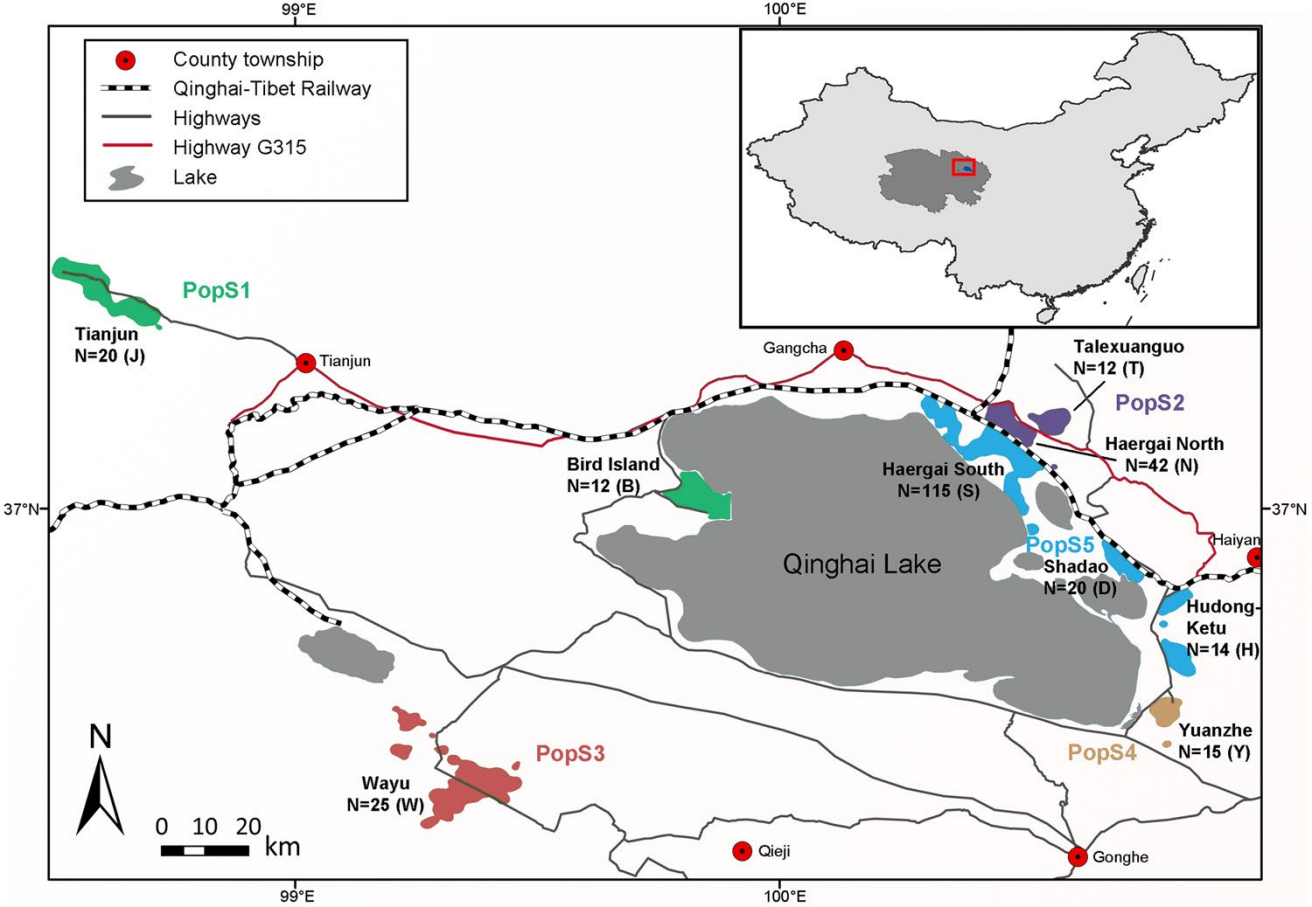
Distribution of all known geographically isolated Przewalski’s gazelle populations of and major anthropogenic infrastructure in the range, including major townships, highways and the Qinghai-Tibet railway. The map was generated using ArcGIS 10.3.1 (ESRI Inc., USA. http://www.esri.com). Population names, abbreviations, and sample sizes from each locality are marked, and population clusters identified by Bayesian population structure inference as implemented in STRUCTURE 2.3.3 are color coded and labeled (PopS1-PopS5).

It is uncertain whether the recently reported increase in the *P. przewalskii* population count was due to actual population recovery or was merely an outcome of improved survey efforts and the discovery of previously unknown populations^6,7^. Overall, human settlements and infrastructure development have exacerbated habitat fragmentation, restricted the movement of most populations, and posed severe threats to the sustainable survival of Przewalski’s gazelle. Among the nine populations known to date, three are located west of Qinghai Lake, including the smallest population, which was not discovered until 2007, on Bird Island (B, n = 19) by the lakeside; Wayu (W, n = 179), which is approximately 50 km southwest^7^; and a distant population in Tianjun (J, n = 282), which is over 100 km northwest of Qinghai Lake. Six populations are located on the east side of Qinghai Lake, namely the Haergai North (N, n = 182), Haergai South (S, n = 340) and Talexuanguo (T, n = 45) populations in Gangcha County and the Shadao (D, n = 113), Hudong-Ketu (H, n = 97) and Yuanzhe (Y, n = 53) populations in Haiyan County (Fig. 1)^7^. Although the eastern Przewalski’s gazelle populations are adjacent geographically, movement and inter-population interactions are likely to be inhibited by major highways, fencing for agriculture, livestock grazing across the region, and more recently, the Qinghai-Tibet railway.

The Qinghai-Tibet railway, or the Qingzang railway, is a high-elevation railway connecting Tibet to other provinces in China. The line cuts through the Qinghai-Tibet Plateau and includes the highest point of all railways in the world (5,072 m at the Tanggula Pass). The construction of the eastern section, which stretches 815 km from Xinning to Golmud and crosses through the terrain of Przewalski’s gazelles in Qinghai, began in 1974 and opened to traffic in 1984. The 1,142 km-long western section between Golmud and Lhasa, however, was not opened until 2006. In October of 2006, a 1.8-m high wired enclosure was built alongside the railway, thus making it impassable for ungulates except by bridges and culverts^8^. The influence of the railway on the local environment and wildlife has been a concern since its construction.

Local economic growth in the recent past may have further intensified the extent of anthropogenic habitat fragmentation in certain areas. Haergai county is home to the largest number of Przewalski’s gazelles, accounting for 40% the species’ total population^9^. This area is also an important hub of the Qinghai-Tibet railway, which runs across the county and divides the gazelles’ range into two subregions, namely the Haergai North (N) and Haergai South (S) regions. There are eight culverts underneath the railway constructed to allow for pedestrian or vehicle traffic, or for water overflow along the 10-km Haergai North-South boundary, in hopes that they might mitigate the negative effects of the railway on wildlife. However, no signs of gazelles crossing these passages have been found in a survey from 2007–2009, thus casting doubt over the effectiveness of these artificial constructions as wildlife corridors^10^. On the basis of these observations that the railway remains an unbridgeable barrier for ungulates, gazelles from Haergai North and Haergai South have been considered as two isolated populations^10^. To the northeast of the railway, the Talexuanguo population is separated from Haergai North by highway G315 (Fig. 1), a national highway and an important regional traffic line that was constructed in 1954^11^. Although highway G315 is wider and carries a larger traffic volume than the Qinghai-Tibet railway, it is not fenced or enclosed, and hence, gazelles from Haergai North have been observed moving across to Talexuanguo in the north^10^. The actual level of genetic connectivity among the populations in Haergai North, Haergai South and Talexuanguo serves as an excellent model to elucidate the crucial factors associated with genetic differentiation and habitat fragmentation in Przewalski’s gazelles.

The effects of roads and railways on increasing habitat fragmentation have long been a focus of research in wildlife conservation^12,13^. As early as 1974, a study showed that roads are effective barriers to small forest mammals^14^, and later ecological and genetic approaches validated such an outcome in other taxonomic groups, including amphibians and insects^15,16^. Recent studies have associated the Qinghai-Tibet railway with an adjustment in the daily activity schemes of the Tibetan antelope (*Pantholops hodgsonii*) and Tibetan gazelle (*Procapra picticaudata*), as well as detours in the migration of Tibetan antelopes to circumvent obstacles^17,18^. Similarly, human settlements, roads and railways have separated Przewalski’s gazelles into small, isolated populations that are likely to be more vulnerable to environmental stochasticity^13,19^. However, the probable genetic consequences of the Qinghai-Tibet railway have not been thoroughly evaluated. Yang *et al*.^11^ have indicated that the most significant factor leading to landscape genetic differentiation among Przewalski’s gazelles is human settlements, such as villages and farms, although a moderate effect of the railway has also been reported.

Population genetic data on Przewalski’s gazelle are scarce, and the first such study was not available until 2003, reporting one mtDNA fragment sequenced in 29 tissue samples from the four populations known at that time^20^. In subsequent years, microsatellite markers were used to identify individuals from fecal samples and to evaluate genetic diversity generally^21,22^. Yang *et al*.^23^ collected samples from most known populations in 2011 and genotyped 13 microsatellite loci to analyze genetic structure from a landscape perspective; yet, information from two critical populations in Wayu and Talexuanguo was missing. Here, we collected specimens from across the geographic range of this species (Fig. 1 and Table 1) and assessed both mtDNA and microsatellite markers to gain a comprehensive understanding of the population genetic structure and diversity of Przewalski’s gazelles. In particular, we examined the fine-scale landscape genetic features of three populations (Haergai North, Haergai South and Talexuanguo) that were delineated by a national highway or railway in the area northeast of Qinghai Lake. The results shed important light on the genetic consequences of how the Qinghai-Tibet railway has led to the fragmentation of habitat for one of the world’s most endangered ungulates.

**Table 1:** Sampled *P. przewalskii* populations in this study and number of samples from each population specifying those succeeding in mtDNA and microsatellite analyses.

Population	2009 Census size^7^	No. Samples	No. Feces	No. Tissues	No. Samples with mtDNA data	No. Samples with microsatellite data
**Bird Island (B)**	19	12	11	1	7	9
**Shadao (D)**	113	20	18	2	16	17
**Hudong-Ketu (H)**	97	14	14	0	10	10
**Tianjun (J)**	282	20	20	0	14	20
**Haergai-North (N)**	182	42	20	22	30	33
**Haergai-South (S)**	340	115	22	93	75	78
**Talexuanguo (T)**	45	12	12	0	12	12
**Wayu (W)**	179	25	25	0	21	13
**Yuanzhe (Y)**	53	15	14	1	10	12
**Total No. Samples**		275	156	119	195	204
**Total No. Individuals**	1,310				181	184

## Results

### Summary statistics for the mtDNA and microsatellite analyses

**Figure 2:**
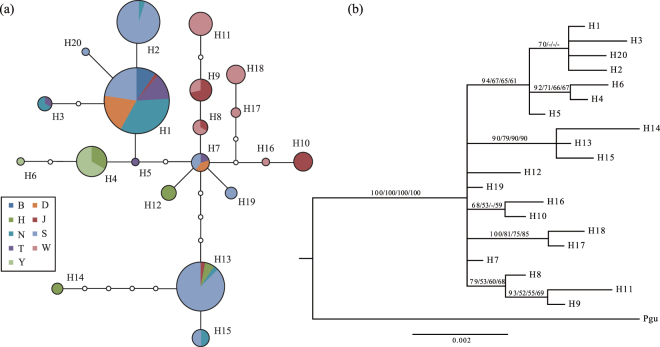
Phylogeography of *P. przewalskii*, on the basis of 1,261 bp of concatenated mtDNA haplotypes, including *CytB*, *12 S* rRNA, and the control region. (**a**) Statistical parsimony network constructed in TCS 1.21, with area of the circles (pie charts) corresponding to the number of individuals sharing this haplotype and the distance between circles proportional to the number of nucleotide substitutions. Colors within the pie chart correspond to different sampling localities (see Fig. 1 for the full name of the populations), and size is proportional to the number of individuals from a particular population sharing this haplotype. Missing haplotypes in the network are represented by small open circles. (**b**) Phylogenetic relationship of mtDNA haplotypes reconstructed with MrBayes and PAUP, with mtDNA sequence of *Procapra gutturosa* (Pgu) as outgroup. Numbers on the branches are bootstrap values supporting the node using Bayesian/maximum likelihood (ML)/maximum parsimony (MP)/Neighbor Joining (NJ) approaches (only those over 50% are indicated).

MtDNA sequences spanning *CytB*, *12 S* rRNA, and the control region were concatenated into 1,261-bp haplotypes and assembled for 181 individuals from 83 tissue samples and 112 fecal samples. These sequences were clustered into 20 haplotypes with 24 polymorphic sites, including 23 SNPs (single nucleotide variations) and one indel (Table 2). The nucleotide diversity (π) of Przewalski’s gazelle was 0.00336 (Table 3), significantly lower than those of closely related taxa, namely the Tibetan gazelle (0.08^24^) and Mongolian gazelle (0.0585^25^). The gene diversity (H) of this species was 0.840, a value comparable with that of the European roe deer (*Capreolus capreolus*), another ungulate species that had been significantly affected by human activities (H = 0.18–1.0, π = 0.0022–0.011^26^). All seven individuals from Bird Island (B) shared one mtDNA haplotype, H1, which was the most prevalent haplotype and was shared by 62 individuals from six populations (Fig. 2a). This flat matrilineal variation may have been the result of a founder effect or demographic bottleneck and was consistent with the population’s isolated locality and smallest census size among all (only 19 individuals were counted as of 2009^7^). Similarly, diminishing mtDNA diversity was also observed in the second smallest population, Yuanzhe (Y, estimated at 53 gazelle as of 2009^7^), consisting of two haplotypes that differed by only 1 bp (H4 and H6, Fig. 2, Tables 2 and 3).

**Table 2:** MtDNA haplotypes and variable sites in *P. przewalskii*.

Locus		Control Region																					*12* *S*		
Primers		PprCR1-1-F				PprCR3-F						PprCR4-F										PprCR5-F	Ppr12S1-F		
		PprCR1-1-R				PprCR3-1-R						PprCR4-1-R										PprCR7-R	Ppr12S2-R		
**Position**		**1**	**1**	**1**	**1**	**1**	**1**	**1**	**1**	**1**	**1**	**1**	**1**	**1**	**1**	**1**	**1**	**1**	**1**	**1**	**1**	**1**			
		**5**	**5**	**5**	**5**	**5**	**5**	**5**	**5**	**5**	**5**	**5**	**5**	**5**	**5**	**5**	**5**	**5**	**5**	**5**	**5**	**6**			
		**4**	**5**	**5**	**6**	**7**	**8**	**8**	**8**	**8**	**8**	**8**	**8**	**9**	**9**	**9**	**9**	**9**	**9**	**9**	**9**	**1**	**7**	**7**	**7**
		**4**	**2**	**5**	**2**	**9**	**0**	**2**	**3**	**4**	**5**	**7**	**8**	**0**	**0**	**1**	**1**	**1**	**2**	**4**	**5**	**4**	**2**	**3**	**6**
**Haplotype**	**N**	**6**	**6**	**9**	**6**	**3**	**2**	**9**	**8**	**9**	**3**	**0**	**4**	**7**	**8**	**0**	**2**	**9**	**6**	**6**	**3**	**3**	**0**	**1**	**8**
**H1**	62	T	A	G	A	A	G	C	T	G	T	G	T	G	A	A	C	C	A	A	G	C	C	A	A
**H2**	21	.	.	.	.	G	.	.	.	.	.	.	.	.	.	.	.	.	.	.	.	.	.	.	.
**H3**	3	.	.	.	.	.	.	.	.	A	C	.	.	.	.	.	.	.	.	.	.	.	.	.	.
**H4**	12	.	.	.	.	.	.	.	.	.	.	.	.	.	.	.	T	.	.	.	A	.	.	.	.
**H5**	1	.	.	.	.	.	.	.	.	.	.	.	.	.	.	.	T	.	.	.	.	.	.	.	.
**H6**	1	.	.	.	.	.	.	.	.	.	.	.	C	.	.	.	T	.	.	.	A	.	.	.	.
**H7**	5	.	.	.	G	.	.	.	.	A	.	.		.	.	.	T	.	.	.	.	.	.	.	.
**H8**	3	.	.	.	G	.	.	.	.	A	.	.	.	.	.	.	T	.	.	.	.	T	.	.	.
**H9**	7	.	.	.	G	.	.	.	.	A	.	.	.	.	.	.	T	.	.	.	.	T	.	.	G
**H10**	6	.	.	.	G	.	.	.	C	A	.	.		.	.	.	T	.	.	.	.	.	.	.	.
**H11**	8	.	.	.	G	.	.	.	.	A	.	.	.	A	G	.	T	.	.	.	.	T	.	.	G
**H12**	3	.	.	.	G	.	.	.	.	A	.	.	.	.	.	G	T	T	.	.	.	.	.	.	.
**H13**	32	C	.	.	G	.	A	T	.	A	.	.	.	.	.	.	T	.	.	G	.	.	.	.	.
**H14**	1	C	.	.	G	.	A	T	.	A	.	.	.	.	.	.	T	.	.	G	.	.	T	G	G
**H15**	4	C	.	.	G	.	A	T	.	A	.	.	.	.	.	.	T	.	.	G	T	.	.	.	.
**H16**	1	.	.	A	G	.	.	.	C	A	.	.	.	.	.	.	T	.	.	.	.	.	.	.	.
**H17**	2	.	.	A	G	G	.	.	.	A	.	A	.	.	.	.	T	.	.	.	.	.	.	.	.
**H18**	6	.	.	A	G	G	.	T	.	A	.	A	.	.	.	.	T	.	.	.	.	.	.	.	.
**H19**	2	.	-	.	G	.	.	.	.	A	.	.	.	.	.	.	T	.	.	.	.	.	.	.	.
**H20**	1	.	.	.	.	.	.	.	.	.	.	.	.	.	.	.	.	.	G	.	.	.	.	.	.

**Table 3:** Genetic diversity of *P. przewalskii* populations on the basis of mtDNA and microsatellite analyses.

Population	MtDNA sequences				Microsatellite markers				
	N	pi	gene diversity	π	H_E_	H_O_	MAR	MAN	F_IS_
**Bird Island (B)**	1	0.000	0	0	0.391 ± 0.197	0.460 ± 0.268	3.22 ± 1.64	2.40 ± 0.84	−0.202
**Shadao (D)**	2	0.791 ± 0.609	0.264 ± 0.136	0.00063 ± 0.00054	0.508 ± 0.215	0.450 ± 0.214	5.30 ± 4.16	3.70 ± 1.42	0.118
**Hudong-Ketu (H)**	4	5.400 ± 2.843	0.778 ± 0.091	0.00428 ± 0.00255	0.478 ± 0.217	0.447 ± 0.212	4.60 ± 2.50	3.40 ± 1.43	0.068
**Tianjun (J)**	5	2.549 ± 1.456	0.725 ± 0.086	0.00202 ± 0.00130	0.556 ± 0.200	0.548 ± 0.182	5.50 ± 2.80	4.60 ± 1.58	0.017
**Haergai-North (N)**	5	1.903 ± 1.119	0.396 ± 0.115	0.00151 ± 0.00099	0.519 ± 0.175	0.517 ± 0.184	7.80 ± 5.43	4.30 ± 1.16	0.004
**Haergai-North (S)**	7	4.078 ± 2.058	0.718 ± 0.030	0.00323 ± 0.00181	0.543 ± 0.202	0.523 ± 0.183	7.50 ± 3.47	5.00 ± 1.70	0.036
**Talexuanguo (T)**	4	1.018 ± 0.736	0.491 ± 0.175	0.00081 ± 0.00066	0.479 ± 0.174	0.495 ± 0.223	4.00 ± 3.71	2.80 ± 0.63	−0.033
**Wayu (W)**	6	4.057 ± 2.108	0.781 ± 0.061	0.00322 ± 0.00187	0.638 ± 0.180	0.571 ± 0.195	5.60 ± 2.32	4.70 ± 1.89	0.110
**Yuanzhe (Y)**	2	0.200 ± 0.269	0.222 ± 0.166	0.00018 ± 0.00026	0.543 ± 0.179	0.488 ± 0.217	3.90 ± 3.69	3.20 ± 1.14	0.109
**Total**	20	4.241 ± 2.114	0.830 ± 0.020	0.003363 ± 0.001855	0.584 ± 0.162	0.512 ± 0.012	10.10 ± 4.51	7.40 ± 1.84	

N: Number of mtDNA haplotypes found in the population, pi: mean number of pairwise differences, π: nucleotide diversity;

H_E_: expected heterozygosity based on microsatellite markers, H_O_: observed heterozygosity based on microsatellite markers;

MAR: mean allele range; MAN: mean allele number; F_IS_: inbreeding coefficient based on microsatellite markers.

The statistical parsimony network and phylogeny revealed similar topology among Przewalski’s gazelle mtDNA haplotypes (Fig. 2). Three haplotypes (H13, H14 and H15) clustered into one clade with strong bootstrap values, and the other 15 clustered into another clade with moderate support. The eastern populations of Haergai North, Haergai South and Hudong-Ketu and the westernmost Tianjun population carried mtDNA haplotypes from both clades, thus suggesting the existence of ancestral polymorphism in Przewalski’s gazelles before their divergence into distinct populations and the retention of such genetic diversity within these relatively large populations (Table 2). In contrast, seven haplotypes (H8–11, H16–18), all within the same cluster (Fig. 2a), were found exclusively in Tianjun and Wayu and accounted for 90% of the individuals in these two populations. This moderate level of matrilineal divergence between the Tianjun and Wayu populations and the rest suggested an overall east-west division across the range of Przewalski’s gazelle, with Tianjun and Wayu in the west and the others in the east (Fig. 1). The mitochondrial phylogenetic placement of the Bird Island population is uncertain, owing to its small sample size, and the mtDNA haplotype that this population contained was found in both the east and west.

Reliable microsatellite genotyping data from at least six out of the ten loci were obtained from 204 samples, including 114 feces (out of 156 samples) and 90 tissues (out of 92 that were tried). The combined unbiased PID value of the ten microsatellite loci was 6.05e-8, thus indicating sufficient power of the panel of markers for individual identification. Combining microsatellite and mtDNA information, we identified 184 individuals, and duplicate samples were removed from the downstream analysis. The sex ratio was evaluated for a subset of 94 individuals, and 41 females and 53 males were recognized. No significant allele dropout or null allele was detected in the final dataset, and after Bonferroni correction, there was no significant linkage disequilibrium between loci. A total of 74 alleles were found with an overall observed heterozygosity (H_O_) of 0.512 and a mean allele range (MAR) of 10.10. In accordance with the mtDNA diversity indices, the microsatellite diversity of the Bird Island population was the lowest among all populations, whereas the Tianjun, Wayu, Haergai North and Haergai South gazelle populations possessed relatively large heterozygosities and allele richness (Table 3).

Significant levels of recent, unidirectional migration were detected in four population pairs, including those in Tianjun to Bird Island, Haergai South to Shadao, Haergai South to Hudong-Ketu, and Haergai North to Talexuanguo. The migration rate of these source populations, which was determined as the proportion of individuals in a population that were emigrants, was over 0.1 (Table 4). Except for Tianjun and Bird Island, which were distant but nevertheless shared a mutual mtDNA haplotype H1, these migration events occurred among geographically proximate populations. Intriguingly, corresponding to their location relative to the Qinghai-Tibet railway, the three inter-connecting populations, Haergai South, Shadao and Hudong-Ketu, are all located to the south of the railway, whereas both Haergai North and Talexuanguo lie to the north (Fig. 1). Segregated by the railway, Przewalski’s gazelle from Haergai North and South had a very low migration rate (0.0683 and 0.0242) despite having the shortest physical distance between them among all known populations. This finding indicated that migration between Haergai North and South was probably blocked by barriers specific to these two populations, and the Qinghai-Tibet Railway was the most likely barrier. The pattern of genetic connectivity among populations within this landscape east of Qinghai Lake suggested a disruption of isolation-by-distance corresponding to the presence of the railway.

**Table 4:** Migration rates among *P. przewalskii* populations estimated in BayesAss 3.0.3, highlighting the significant level of pairwise gene flow in four population pairs.

Pop-out	B	D	H	J	N	S	T	W	Y
Pop-in									
**B**		0.0172	0.0175	**0.1771**	0.0201	0.0216	0.0172	0.0177	0.0204
**D**	0.0120*		0.0126	0.0228	0.0583	**0.1673**	0.0129	0.0131	0.0143
**H**	0.0157	0.0155		0.0212	0.0329	**0.1799**	0.0149	0.0152	0.0169
**J**	0.0122	0.0132	0.0120		0.0251	0.0263	0.0137	0.0138	0.0142
**N**	0.0087	0.0084	0.0083	0.0116		0.0683	0.0086	0.0095	0.0091
**S**	0.0048	0.0056	0.0048	0.0070	0.0242		0.0050	0.0048	0.0051
**T**	0.0136	0.0141	0.0138	0.0132	**0.2160**	0.0174		0.0135	0.0137
**W**	0.0155	0.0187	0.0155	0.0304	0.0277	0.0210	0.0152		0.0214
**Y**	0.0164	0.0172	0.0164	0.0178	0.0440	0.0404	0.0174	0.182	

^*^Numbers represent the proportion of individuals migrating from “pop-out” to “pop-in”.

### Population genetic structure analysis

Mitochondrial DNA-based AMOVA analysis provided support for two groups of Przewalski’s gazelles corresponding to mtDNA phylogeography (Fig. 2), one with Tianjun and Wayu in the west and another with all other populations in the east (F_ST_ = 0.4606, *p* < 0.05, Table 5). In addition, the population pairwise F_ST_ calculated on the basis of mtDNA haplotypes also indicated a strong landscape genetic structure, in which Tianjun, Wayu, Hudong-Ketu, Haergai South and Yuanzhe were the most distinct populations, and each of these populations significantly differed from the others (Table 6).

**Table 5:** Measures of different scenarios of geographic division of *P. przewalskii* populations, on the basis of AMOVA with mtDNA and microsatellite data.

Subdivision	mtDNA F_ST_	Microsatellite F_ST_
**(J-W) (B-N-T-S-D-H-Y)**	**0.4606**	0.07174
**(J) (W) (B-N-T-S-D-H-Y)**	0.4494	0.07229
**(J-B) (W) (N-T-S-D-H-Y)**	0.4104	0.07201
**(J-B) (W) (N-T) (S-D-H-Y)**	0.3631	**0.07304**
**(J-B) (W) (N-T) (S-D-H) (Y)**	0.3649	0.07141

^*^For all F_ST_ values, *p* < 0.05, indicating significant difference.

**Table 6:** Genetic differentiation between all sampled populations of *P. przewalskii* as measured by pairwise mtDNA (above the diagonal) and microsatellite (below the diagonal) F_ST_.

F_ST_	B	D	H	S	T	N	Y	J	W
**B**	—	−0.012	0.366*^1^	0.244*	−0.007	−0.027	0.936*	0.610*	0.555*
**D**	0.16608*	—	0.385*	0.229*	−0.070	−0.012	0.751*	0.574*	0.554*
**H**	0.12933*	0.04705*	—	0.120*	0.336*	0.352*	0.316*	0.199*	0.295*
**S**	0.02799	0.09677*	0.05460*	—	0.215*	0.185*	0.389*	0.283*	0.373*
**T**	0.03322	0.07070*	0.03493	0.04822*	—	−0.027	0.715*	0.537*	0.525*
**N**	0.08950*	0.10515*	0.05645*	0.06493*	0.00747	—	0.534*	0.519*	0.552*
**Y**	0.07368*	0.13148*	0.11687*	0.03952*	0.09698*	0.13201*	—	0.651*	0.585*
**J**	0.05024*	0.06263*	0.01571	0.01957*	0.01378	0.04576*	0.05564*	—	0.190*
**W**	0.14804*	0.05772*	0.09002*	0.13553*	0.07031*	0.11818*	0.14319*	0.06124*	—

^1^ F_ST_ values with *p* < 0.05 are marked with *indicating significant difference.

**Figure 3:**
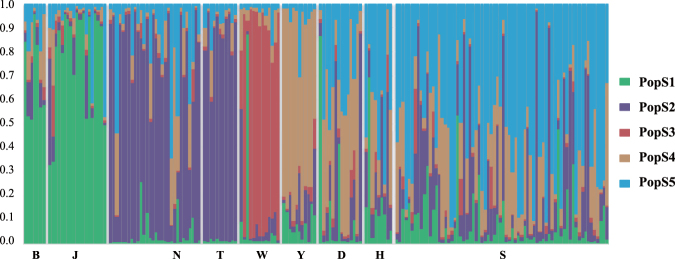
Bayesian population structure analysis of 184 Przewalski’s gazelles, on the basis of microsatellite genotyping data estimated by STRUCTURE 2.3.3. Simulations were run for 1,000,000 iterations after a 100,000 burn-in under the model with an admixture ancestry and uncorrelated frequency and K = 5 as the highest posterior probability among other choices of K. Each individual is represented by a thin vertical line (X axis), which is partitioned into five colored segments that represent the individual affiliation to each of the five clusters. Different colors and Y axis represent the proportion of each individual belonging to different putative populations, as noted in the figure.

The population structure analysis based on the microsatellite genotyping data revealed fine-scale partitioning of genetic variation at the landscape level. Microsatellite-based AMOVA analysis supported the strongest geographic partitioning into four subpopulations (F_ST_ = 0.07304, *p* < 0.05, Table 5) while the Bayesian clustering approach implemented in STRUCTURE suggested a division of the 184 Przewalski’s gazelles into five groups (PopS1-S5, Table S4, Figs 1, 3). PopS1 predominantly consisted of individuals from Bird Island and Tianjun, PopS2 from Haergai North and Talexuanguo, PopS3 from Wayu, PopS4 from Yuanzhe, and PopS5 from Haergai South, Shadao and Hudong-Ketu. Genetic admixture was detected between the PopS5 populations of Haergai South, Shadao and Hudong-Ketu and the PopS4 population of Yuanzhe, all located south of the Qinghai-Tibet railway, and this finding was consistent with the high migration rate among these populations, as detected in BayesAss (Table 4). The other two pairs of interconnected populations with strong migration rates detected, Bird Island vs. Tianjun and Haergai North vs. Talexuanguo, were also supported as belonging to PopS1 and PopS2, respectively. Intriguingly, to the north of the Qinghai-Tibet railway, Haergai North clustered with Talexuanguo, forming a population (PopS2) in Hardy-Weinberg Equilibrium. In contrast, despite the geographic proximity between Haergai North and South, the Haergai South population was genetically more closely associated with those in Hudong-Ketu (PopS5) and Yuanzhe-Shadao (PopS4), all of which are located to the south of the Qinghai-Tibet railway. The genetic distance between PopS2 and PopS5 was larger than that between PopS4 and PopS5 (Table S5). This landscape population genetic structure further suggested that the Qinghai-Tibet railway may have contributed to the genetic substructure of Przewalski’s gazelles by impeding gene flow across the railway. The three populations west of Qinghai Lake were recognized as two populations, PopS1 (Bird Island and Tianjun) and PopS3 (Wayu). The genetic association of the Bird Island population with that of Tianjun in the microsatellite-based analysis assigned Bird Island to the western group of Przewalski’s gazelle and hence resolved its matrilineal phylogenetic ambiguity. All but one individual from Wayu belonged to PopS3, which appears to be the most distant population based on allele frequency differentiation (Table S5), highlighting the genetic uniqueness of Wayu population. The only non-PopS3 individual from Wayu was assigned to PopS1, thus suggesting genetic connectivity between Tianjun and Wayu to a certain extent and supporting the overall east-west divergence across the range of Przewalski’s gazelle.

**Figure 4:**
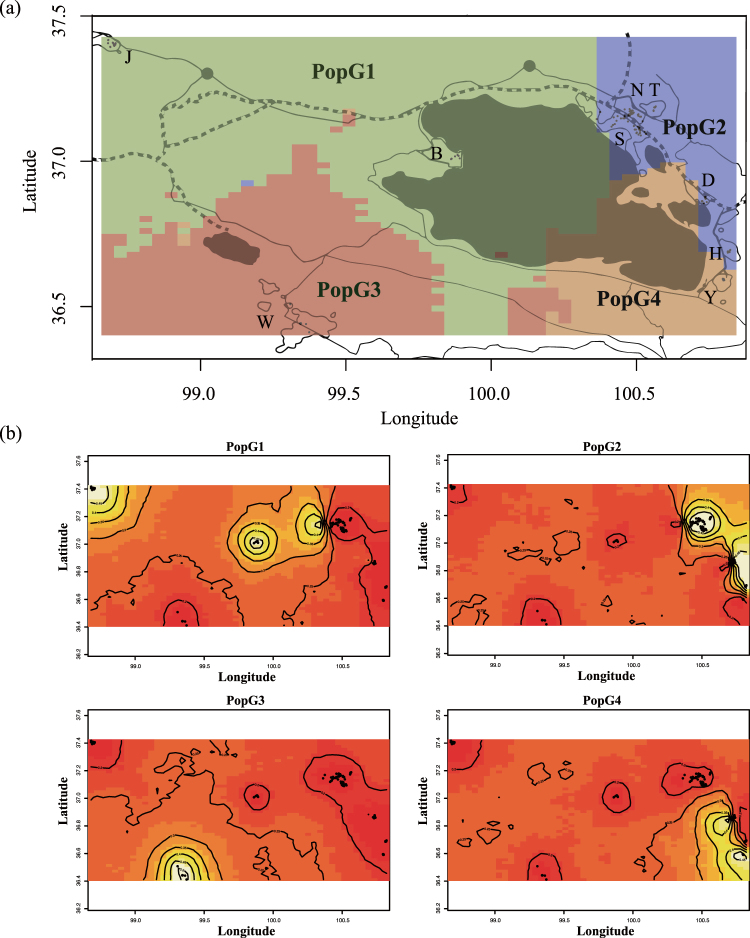
Spatial genetic groups estimated by GENELAND with an uncorrelated model and 1,000,000 iterations when K = 4. (**a**) Cluster membership showing geographic information, including distribution areas of Przewalski’s gazelles, roads and the railway. The map was generated using ArcGIS 10.3.1 (ESRI Inc., USA. http://www.esri.com). (**b**) Posterior probability of individuals belonging to each of the four groups showing geographic distribution of individuals, with lighter colors indicating higher probability.

GENELAND’s Bayesian algorithm incorporated geographic information with the microsatellite genotyping data and clustered our sampled Przewalski’s gazelles into four putative groups (PopG1-G4, Fig. 4, Figure S2). The PopS1, PopS3 and PopS4 groups identified in STRUCTURE corresponded to PopG1, PopG3 and PopG4 in GENELAND, respectively, and the other five populations (Haergai North, Haergai South, Talexuanguo, Shadao and Hudong-Ketu) that mostly fell into PopS2 and PopS5 in STRUCTURE were designated a single cluster PopG2 in GENELAND (Fig. 4 and Table S4). These five populations are geographically close to one another, and all are located on the northeastern side of Qinghai Lake. The different population clustering results between STRUCTURE and GENELAND were likely to have been due to the consideration of geographic information in GENELAND, whereas the recent genetic divergence detectable in Structure was not sufficiently strong to overcome the influence of spatial proximity.

### Demography and population divergence modeling

To further explore the potential effect of the railway on gene flow and population divergence over a short period of time, particularly for the Haergai North and South populations, we simulated microsatellite genotyping datasets for two hypothetical populations that have undergone isolation under four different demographic scenarios varying in terms of the duration of isolation and the existence of migration (Table 7). The pairwise genetic distance measure (F_ST_) was used as an indicator of the outcome of different demographic variables on population divergence. A strong correlation was detected between the maintenance of migration and inter-population genetic connectivity. If gene flow could be maintained through migration between the two populations, the level of population differentiation would remain relatively low after 20 generations of separation (F_ST_ = 0.0185, Model 1) and would remain approximately the same even after another 30 generations (F_ST_ = 0.0192, Model 3). However, if gene flow were blocked, the average F_ST_ would increase to 0.0347 after only five generations (Model 2) and would further increase to 0.1119 after 35 generations (Model 4). Model 2 recapitulated the scenario of Haergai North and South Przewalski’s gazelles at present, in which the two populations have been delineated by the Qinghai-Tibet railway since the 1970s (20 generations ago); however, migration across the railway remained possible until the late 2000s (5 generations ago) when a 1.8 m high enclosure was wired alongside the railway, thus creating a barrier to the passage of wild ungulates. This simulation results suggest the significance of migration, even at low levels (the proportion of migrants in the Haergai North or Haergai South population from the other populations is estimated at 0.0242–0.0683, Table 4), in maintaining population connectivity. This simulation supports that the enclosed Qinghai-Tibet railway may have effectively blocked gene flow between Haergai North and Haergai South, thus resulting in a detectable level of genetic differentiation after as few as five generations. Such effects of habitat fragmentation and genetic divergence would continue and become more significant in the future, as suggested by Model 4, if no facilitation of inter-population migration were to be implemented.

**Table 7:** Genetic differentiation (F_ST_) of two simulated *P. przewalskii* populations in fastsimcoal 2.5.2.21 under different demographic models of divergence and migration.

	Time* of separation	Time since migration being blocked**	Average F_ST_***
**Model 1 (present)**	20	0	0.0185
**Model 2 (present)**	20	5	0.0347
**Model 3 (future)**	50	0	0.0192
**Model 4 (future)**	50	35	0.1119

^*^Time is presented in unit of generation and a generation time of two years is used. Models 1 and 2 represent the current time point when population separation occurred 20 generations ago since the construction of Qinghai-Tibet railway in the 1970s. Models 3 and 4 project the scenario in 60 years from present.

**Migration between the two populations was set to constantly occur in Models 1 and 3, while in Models 2 and 4, migration between populations was set to stop 15 generations after the initial isolation, corresponding to the construction of fence along the Qinghai-Tibet railway around 2006.

***Population differentiation is calculated in Arlequin 3.5 based on 1,000 permutations.

## Discussion

We conducted the most recent population genetic analysis of Przewalski’s gazelles, with a concatenated 1,261 bp of mtDNA sequences and the genotypes of 10 microsatellite loci from nearly 300 specimens collected across the range of this species. Considering the total population size of 1,300–1,600 for the entire species^6,7^, this sampling is representative and sufficient for a comprehensive assessment of the species’ genetic variability. The overall low mtDNA diversity in the Przewalski’s gazelle may reflect its low historical effective population size owing to the Pleistocene climatological changes, environmental dynamics on the Qinghai-Tibet Plateau and/or the severe anthropogenic-driven population decline in the past century. Nevertheless, this low diversity suggests that although new populations have been discovered at sites where the gazelle has not been previously reported, thus leading to its reclassification on the IUCN Red List from Critically Endangered to Endangered, the survival of Przewalski’s gazelle remains a pressing issue. Conservation measures should be taken immediately to prevent further loss of genetic diversity due to habitat fragmentation and the interruption of dispersal among populations.

It has been demonstrated that human settlement is a major factor contributing to the landscape genetic differentiations currently observed in Przewalski’s gazelles^10^. Here, combining population structure and migration analyses, we also associated the genetic structure of gazelle populations across the range with recent anthropogenic activities. Przewalski's gazelles in Yuanzhe have been recognized previously as an isolated population^21,22^, which was also verified as a unique clade in both STRUCTURE (PopS4) and GENELAND (PopG4) in this study (Figs 3 and 4). Although Yuanzhe is geographically close to Hudong-Ketu, and there is no major highway or railway between these regions, gazelles there have been physically blocked from the Hudong-Ketu population by a large ranch with thousands of heads of livestock and related human settlements since 1966^7,10^. These population genetic patterns suggest a strong influence of human settlements on decreasing gene flow and suggest that such process could be rapid, thus leading to significant, detectable population genetic divergence after merely half a century.

Our study also provides the first evidence of an even more recent genetic consequence of the Qinghai-Tibet railway on Przewalski’s gazelle’s landscape genetic pattern. The railway across the Haergai area was open to traffic in the 1980s but was probably not a barrier to ungulate movement until 2006, when wired fences were built along the railroad, thus making it impassable for large mammals. This period of time was roughly equivalent to five generations of isolation at most, yet a moderate level of genetic divergence between the railway-divided Haergai North and South gazelle populations was already detectable in the clustering analysis implemented in STRUCTURE (Fig. 3). The migration rate between the two populations was low compared with that of other geographically neighboring populations (Table 4). Intriguingly, the populations of Haergai North and Talexuanguo, both located on the northern side of the railway, grouped together with a significant extent of gene flow (PopS2 in STRUCTURE analysis). The same pattern was observed on the southern side, where gazelles of the Haergai South, Hudong and Shadao populations were identified in STRUCTURE as the belonging to the same cluster (PopS5), and a considerable level of genetic connection was evident. Measurements of the genetic differentiation between two hypothetical populations simulated under various demographic models also highlighted the important role of gene flow in counteracting population fragmentation. With a low migration rate, the level of genetic divergence between geographically separated populations would remain relatively low for decades, whereas two isolated populations without any migration would differentiate significantly after as few as five generations (Table 7). These simulated models corroborated our real-world data, thus indicating that the Qinghai-Tibet railway may have interrupted gene flow and effectively separated the originally continuous Przewalski’s gazelle population in Haergai into two distinct groups on either side of the railway.

The genetic divergence between the Haergai North and South populations was in accordance with field studies that no gazelle passage was detected crossing the culverts underneath the Qinghai-Tibet railway^10^. It is noteworthy though no genetic differentiation between Haergai North and South gazelles was reported prior to the enclosure of Qinghai-Tibet railway in this area based on samples collected in 2006^23^. By contrast, gazelles in Haergai North have been observed moving alongside the railway to Talexuanguo^10^, consistent with the genetic connectivity evident from population structure and migration analyses. Therefore, in the area of Haergai and Talexuanguo, multiple lines of evidence converged on the conclusion that the 1.8-meter wired enclosure along the railway, which was constructed less than a decade prior, may have become an effective obstacle keeping the gazelles from crossing. The gazelles’ avoidance of the culverts beneath the railway may be because these tunnels are too narrow for ungulates and/or are frequently occupied by humans^27,28^. Nevertheless, because artificial passage through the railway is the only available option for facilitating movement and alleviating the isolation between Haergai North and South populations, conservation measures such as building wildlife-friendly corridors over the Qinghai-Tibet railway and the reduction of human disturbance are recommended.

In addition to railways, highways represent other anthropogenic landscape infrastructure features contributing to the fragmentation of the habitat of Przewalski’s gazelles^11^. To the north of the Qinghai-Tibet railway, the small gazelle population in Talexuanguo, with approximately 60 individuals^6^, is separated from that in Haergai North by a national highway, highway G315. This highway is wider, busier and was open to traffic earlier than the Qinghai-Tibet railway. However, with a medium traffic load and without a fenced enclosure to date, the open highway G315 seems to be less of a barrier to ungulates relative to the railway, and Przewalski’s gazelles have occasionally been seen moving across this highway^11^. Genetic analysis also suggested the existence of moderate gene flow between the two populations, which were consistently combined into one single population in the STRUCTURE and GENELAND analyses. However, with the overall economic development in the region, concerns remain over the potential intensified habitat fragmentation that may eventually lead to population-wide genetic differentiation. Long-term and routine field and genetic studies of the Talexuangguo and Haergai North populations are therefore necessary to monitor the status of gene flow and connectivity via highway G315, with particular attention to the maintenance of the highway for the passage of wildlife.

The gazelle population in Wayu, which is southwest of the Qinghai Lake, was not known to the public until 2007^6^, and this is the first study to assess its genetic diversity in relationship to that of other populations. The origin of the Wayu population is unclear, yet it is close to Qieji (approximately 80 km east of Wayu), where a population used to live but was not found during a survey in 2008^10^. Some researchers thus suspected that the Wayu population might be the same group of gazelles that once occupied Qieji and that the population dispersed westward in approximately 2007^10^. One study including samples from the Qieji population in 2011 identified this population as a distinct group in a microsatellite-based population structure analysis^23^, a result similar to the unique status found in the Wayu population in our study (PopS3 in STRUCTURE and PopG3 in GENELAND). However, because microsatellite genotyping data cannot be combined without calibration, we were not able to evaluate whether the Wayu and Qieji populations were indeed the same population or two subpopulations derived from a large historical population that already became differentiated from other populations. The Wayu population has a large census size (ranking fourth out of nine populations^7^) and possessed high genetic diversity relative to the other populations in terms of both mtDNA and microsatellite markers. However, the conservation outlook of the population is not optimistic, because the construction of a national expressway, the G6 from Beijing to Lhasa, has progressed to this region and opened to traffic since 2011. The Wayu section of the G6 cut through the gazelle’s habitat and divided it into two small isolated patches (Fig. 1) without any artificial passages designated for wildlife crossing. The long-term genetic and ecological consequences of the expressway on the Wayu gazelle population, in particular whether the expressway would further intensify the landscape genetic structure and population fragmentation, require close monitoring and attention.

In conclusion, with both maternal and biparental genetic markers, we observed rather low genetic diversity and strong phylogeographic structure for Przewalski’s gazelles across the full geographic range of this species in Qinghai. Overall, there is a strong division between gazelles on the eastern vs. western side of Qinghai Lake, and the nine patches of gazelles can be further clustered into five populations, including the Tianjun/Bird Island (PopS1) and Wayu (PopS3) populations in the west and the Haergai South/Shadao/Hudong-Ketu (PopS5), Haergai North/Talexuanguo (PopS2), and Yuanzhe (PopS4) populations in the east. Our study provides the first evidence of the recent genetic divergence between the Haergai North and Haergai South gazelle populations, corresponding to the construction of the enclosure along the Qinghai-Tibet railway less than ten years ago, an equivalent of five generations. Such a visible genetic impact of habitat fragmentation on a migratory ungulate species like the Przewalski’s gazelle is alarming and highlights the need for wildlife-friendly passages along artificial infrastructure. Well-designed wildlife corridors across the railway and other major highways in this area are critical to alleviating habitat fragmentation and facilitating gene flow. Long-term monitoring of the effects of anthropogenic habitat fragmentation across the range of Przewalski’s gazelles and conservation measures are recommended to avoid further population differentiation and the loss of genetic diversity.

## Materials and Methods

### Biological sampling

During 2010–2012, 156 fecal and 119 tissue samples were collected from all nine Przewalski’s gazelle distribution areas (Fig. 1 and Table 1), including Tianjun (J), Wayu (W), Bird Island (B), Haergai North (N), Haergai South (S), Talexuanguo (T), Hudong-Ketu (H), Yuanzhe (Y) and Shadao (D). We first interviewed local villagers and conservationists about general sightings, identified the regions likely to be occupied by gazelle populations at the time, and traveled to the regions at dawn to locate the herds at their overnight grounds. Right after the animals departed for grazing, we arrived at the nesting area to collect fresh feces and preserved the fecal samples in 95% ethanol. Tissue samples were taken from deceased animals found by local people and were then dried after collection. Samples were transported at ambient temperature. Upon arrival at the laboratory, the ethanol was removed, and all samples were frozen at −80 °C for long-term storage.

Each fecal sample was extracted at least twice to ensure the accuracy of the microsatellite genotyping by using a modified phenol-chloroform method^29^, a QIAamp DNA stool mini kit (Qiagen, USA), or a BioTeke Stool DNA rapid extraction kit (BioTeke Corporation, China). Tissue samples were extracted with a standard phenol-chloroform method. The DNA concentrations and quality were examined with a Nanodrop 2000 spectrophotometer, and the samples were diluted to a working concentration for the downstream PCRs.

### MtDNA, sexing and microsatellite analysis

Six primer sets, amplifying partial regions of mitochondrial *CytB*, *12 S* rRNA, and the control region of *P. przewalskii*, were used in this study. These sets were either modified from those published for other Bovidae species^30,31^ or designed from a conspecific mtDNA sequence (GenBank accession number GU386355) using Primer3^32^ (Table S1). PCR was conducted with a total reaction volume of 25 µL that contained 1X PCR Buffer, 1.5 mM MgCl_2_, 0.8 mM dNTPs, 1 unit of Takara Taq (Takara Bio Inc., Japan) or AmpliTaq Gold DNA polymerase (Thermo Fisher Scientific Inc., USA), 1 µM forward and reverse primers, and 10–25 ng of genomic DNA. For the amplification of fecal samples, 10 µg of BSA (bovine serum albumin) was added to the system to reduce the effect of potential inhibitors. The PCR amplification was performed in a GeneAmp PCR System 9700 thermal cycler (Thermo Fisher Scientific Inc., USA) under the following conditions: denaturation at 94 °C for 3 min for Takara Taq or 10 min for TaqGold; a touchdown process of 94 °C for 30 s, 60–50 °C for 30 s with a decrease of two degrees every two cycles for each annealing temperature, and 72 °C for 45 s; followed by 35 cycles of 94 °C for 30 s, 50 °C for 30 s, and 72 °C for 45 s; and a final extension step of 72 °C for 10 min. The sequences were inspected in Sequencher 5.1, and the concatenated mtDNA haplotypes was aligned for downstream analysis. Measures of population genetic variation, such as the mean number of pairwise differences between populations, nucleotide diversity, and gene diversity, were estimated in Arlequin 3.5^33^.

A sex-differentiation marker AMELX/Y^34^ was amplified under the same PCR conditions, and the sex of an individual was determined on the basis of agarose gel electrophoresis, in which two bands were present for males, one band each for the X (262 bp) and Y chromosomes (217 bp), or only one band was present for females.

Ten microsatellite markers were used for *P. przewalskii* (Table S2), including four that were originally designed for the species^22,23^ and six that were redesigned on the basis of homologous sequences from related species (*Gazella granti* and *Capra hircus*)^35,36^. HEX with the M13 sequence was linked to the forward primers as a florescent label. PCR was performed at a volume of 10 µL, and each contained 1X PCR Buffer, 2 mM MgCl_2_, 1 mM dNTPs, 0.4 unit of Takara Taq DNA polymerase (Takara Bio Inc, Japan), 1 µM primer mix with the forward primer linked to a M13 sequence and fluorescently labeled with HEX, ~25 ng genomic DNA and 10 µg of BSA (bovine serum albumin) for fecal samples. The PCR program began with a denaturation step at 94 °C for 3 min; a touchdown process with 94 °C for 15 s, 60–50 °C for 30 s with a decrease of two degrees every two cycles for each annealing temperature, and 72 °C for 45 s; 35 cycles of 94 °C for 15 s, 50 °C for 30 s and 72 °C for 45 s; and a final extension at 72 °C for 30 min. Microsatellite allele patterns were size fractionated in an ABI 3730xl DNA analyzer (Thermo Fisher Scientific, Inc., USA) and analyzed using GeneMapper 4.1 and Allelogram 2.2.

To ensure genotyping reliability from the potentially degraded fecal DNA samples, a series of multi-tube measures were applied in which DNA extraction was performed at least twice for each sample, and each DNA extract was amplified at least twice for each marker. Thus, a total of four PCRs were conducted for each sample at each locus. We considered an animal to be heterozygous at a locus on the basis of consistent results from at least two duplicates and a homozygote from at least three replicates. Genotypes from different samples were considered to represent the same individual when mtDNA haplotypes were the same and all alleles at all microsatellite loci were identical or when one allele differed by one repeat unit at one locus only. The estimates of genetic variation and tests of genotyping errors and deviation from Hardy-Weinberg equilibrium or linkage equilibrium were carried out using Micro-Checker 2.2.3^37^ and Genepop 4.2.1^38^. To quantify the power of genetic individualization in the panel of microsatellite markers, we computed in Gimlet 1.3.3^39^ the Probability of Identity (PID) value of these ten loci, which is defined as the overall probability that two individuals drawn at random from a given population share identical genotypes at all typed loci. The measures of microsatellite genetic variations in terms of the average number of alleles per locus, average allele size range per locus, average observed heterozygosity and expected heterozygosity were calculated using Arlequin 3.5 and FSTAT 2.9.3.2^40^.

### Phylogenetic and population structure analysis

Phylogenetic relationships among *P. przewalskii* mtDNA haplotypes were reconstructed with four approaches, and the Mongolian gazelle (*P. gutturosa* accession: JN632689.1) was used as outgroup. Jmodeltest 0.1.1^41^ was applied to select the best substitution model fit for the dataset. A Maximum likelihood (ML) analysis with HKY (Hasegawa, Kishino and Yano) + I (with proportion of invariable sites) model, a maximum parsimony (MP) analysis with heuristic search and random addition of taxa, and a neighbor-joining (NJ) tree constructed from Kimura two-parameter distances followed by a branch-swapping procedure were performed in PAUP 4.0^42^, with the reliability of each node in the topology assessed by 100 bootstrap replicates. A Bayesian approach was performed with MrBayes 3.2.1^43^, and the analysis consisted of two parallel MCMC (Markov chain Monte Carlo) runs for 500,000 generations sampled every 500 generations. The migration rate between each population pairs was assessed by BayesAss 3.0.3^44^, which estimates recent migration rates using MCMC. In addition, a statistical parsimony network of mtDNA haplotypes was constructed in TCS 1.21^45^ to infer phylogeographical relationships.

Assessments of pairwise population genetic differentiation and measures of different scenarios of geographic division in Przewalski’s gazelle populations were evaluated by F_ST_ with mtDNA haplotypes (pairwise difference) and microsatellite genotypes using AMOVA, as implemented in Arlequin 3.5. Statistical significance was tested with 10,000 permutations.

The population structure of Przewalski’s gazelles was inferred with Bayesian clustering methods implemented in STRUCTURE 2.3.3^46,47^ and the GENELAND package in R 2.15.1^48,49^. In STRUCTURE, models of all combinations of the ancestry models and the allele frequency models were tested, and an admixture ancestry model with uncorrelated frequency was selected, with a burn-in period of 100,000 and 1,000,000 MCMC repetitions after burn-in. The number of population clusters (K) was set from two to nine, and two parallel runs were performed for each value of K. The simulated likelihood of these runs was summarized with STRUCTURE HARVESTER 0.6.93^50^ (http://taylor0.biology.ucla.edu/structureHarvester/#) to infer the best-fit number of population clusters (Figure S1 and Table S3). GENELAND was run by using both the microsatellite genotyping data and spatial GPS locality information. Samples without GPS records were excluded from this analysis. The model with uncorrelated allele frequency and a null allele parameter was adapted with 1,000,000 iterations, the first 10% of which were discarded as burn-in. The number of clusters was set from one to nine, and the optimal cluster number was selected according to posterior possibilities (Figure S2).

### Genetic modeling of demography and population divergence

To evaluate the potential effect of the Qinghai-Tibet railway as a barrier to gene flow among Przewalski’s gazelle populations, we generated microsatellite genotype datasets for two isolated populations in fastsimcoal 2.5.2.21^51^, a coalescent simulator of genetic diversity under arbitrary evolutionary or demographic scenarios. For each simulated model, the pairwise population difference (F_ST_) was calculated to evaluate the level of divergence under the specific set of demographic parameters. Four scenarios were set with two different lengths of divergence, each with or without migration between populations. Model 1 and Model 2 represented the Haergai North and Haergai South Przewalski’s gazelle populations at present, which have been separated for 20 generations, or 40 years, corresponding to the construction of Qinghai-Tibet railway in the 1970s. Model 3 and Model 4 projected 30 more generations into the future, or a total isolation length of 50 generations. Because the railway may have become an apparent barrier to ungulate migration only after the railway was upgraded with a fenced enclosure in the 2000s, we set the reduction or elimination of migration 15 generations, or 30 years, after the initial separation, corresponding to 5 and 35 generations of isolation in Model 2 and Model 4 respectively. For Model 1 and Model 3, a constant level of migration between populations was allowed (Table 7). In each model, the effective population size (N) of the two simulated populations was set to 100 and 200, with 20 and 40 gene copies sampled from each, respectively, at ten unlinked microsatellite loci. The migration matrix between the two populations was based on the migration rates between Haergai North and South estimated in BayesAss (Table 4). The mutation rate of the microsatellite markers was set at 0.003, which was calculated on the basis of the expected heterozygosity of the Haergai North and Haergai South populations using He = 1 − (1 + 8Nu)^−1/2^ ^52^. We performed 1,000 permutations for each model and calculated the pairwise F_ST_ between the simulated populations in Arlequin 3.5.

### Accession codes

The GenBank accession numbers of the mtDNA sequences discussed in the paper are KP856162-KP856177.

## Electronic supplementary material

Supplementary Information
