# Supplementary material for: Effects of the Qinghai-Tibet Railway on the Landscape Genetics of the Endangered Przewalski’s Gazelle (Procapra przewalskii)
Source: Sci Rep. 2017 Dec 21;7:17983. doi: 10.1038/s41598-017-18163-7 (PMC5740140; doi:10.1038/s41598-017-18163-7)
Supplement: Supplementary file 1 — Supplementary Information [file 41598_2017_18163_MOESM1_ESM.pdf]

Effects of the Qinghai-Tibet Railway on the Landscape Genetics of the Endangered Przewalski's Gazelle (*Procapra przewalskii*)

He Yu, Shiya Song, Jiazi Liu, Sheng Li, Lu Zhang, Dajun Wang & Shu-Jin Luo

Supplementary Information

Supplementary Table S1. Primers used for mtDNA amplification and sex identification in *P. przewalskii*.

Supplementary Table S2. Primers and summary statistics of microsatellite genetic variation in *P. przewalskii*.

Supplementary Table S3. Likelihood of simulations in STRUCTURE 2.3.3 as inferred by STRUCTURE HARVESTER 0.6.93 under different choices of cluster number (K).

Supplementary Table S4. Assignment probabilities of each *P. przewalskii* patch to each of the clusters estimated in STRUCTURE (K=5) and the corresponding assignment results in GENELAND (K=4).

Supplementary Table S5. Population pairwise genetic distance estimated by STRUCTURE 2.3.3, on the basis of differences in microsatellite allele frequency.

Supplementary Figure Legends

Supplementary Figure S1. Simulation likelihood and related parameters in STRUCTURE 2.3.3 with different values of K (from 2 to 10), for selecting the number of clusters with the highest statistical support. The four panels show the relationship between K value and (a) likelihood, (b) first derivative of likelihood, (c) absolute value of second derivative of likelihood, and (d) DeltaK, calculated by the Evanno method in STRUCTURE HARVESTER 0.6.93.

Supplementary Figure S2. Probability distribution of cluster number with 900,000 iterations after a 100,000 burn-in, indicating that K=4 produced the highest probability among all choices of K in GENELAND.

Table S1. Primers used for mtDNA amplification and sex identification in *P. przewalskii*.

Locus	Primer name	Primer sequence (5'-3')	Length (bp)	Reference
CytB	PprCytB2-F	ACAGGCCTATTCCTAGCAAT	200	Modified from *P. guttorosa* [25]
	PprCytB1-R	CAGAAGGATATTTGTCCTCA		
12S	Ppr12S2-F	TGCTTAGCCCTAAACACAAA	253	Modified from *P. guttorosa* [25]
	Ppr12S2-R	TCCTCCTTTGGTTATTAGTTTCA		
Control Region	PprCR1-1-F	CTATCAACACCCAAAGCTGA	253	This study
	PprCR1-1-R	ATGTTTGTGGTCCATGATGT		
	PprCR3-F	CACATGGGTAGGTACAATTA	247	This study
	PprCR3-1-R	ATGGTCATTAAGCTCGTGAT		
	PprCR4-F	TTAAGTGATATTCCTCATGC	295	This study
	PprCR4-1-R	GATTAGCCATTAGTCCATCG		
	PprCR5-F	GGTAGCTATTTAATGAACTT	201	This study
	PprCR7-R	CAGTTAAGTCCAGCTACAAT		
AMELX/Y	SE47	CAGCCAAACCTCCCTCTGC	262/217	[29]
	SE48	CCCGCTTGGTCTTGTCTGTTGC		

Table S2. Primers and summary statistics of microsatellite genetic variation in *P. przewalskii*.

Locus	Primer sequence (5'-3")	Repeat unit	N	Size range (bp)	AR	H_E	H_O	PIC	Reference
SSR01	F:CCCTAGGAGCTTTCAATAAAGAATCGG R:CGCTGCTGTCAACTGGGTCAGGG	(TC)n(CA)n	11	152-174	4.10	0.839	0.6647	0.8161	L01535 [22]
SSR07	F:TGGTGAATGGTGCTCTCATACCAG R:ACGCCAGCAGCCTCTAAAGGAC	(TG)n	6	111-151	2.20	0.5405	0.6111	0.433	BV681688 [22]
test05	F:GCTACCATCAAAAATGTTCC R:GTGTTTTACCGAGCAGTCTC	(CA)n	4	177-191	2.21	0.4848	0.3943	0.4177	This study, based on AF533521 [31]
test08	F:GGTGGCAGGACTGAGCAAGT R:AAAGTGGAAGATTGAAGCAA	(CA)n	7	128-160	2.14	0.4032	0.4262	0.373	This study, based on AF533517 [31]
test16	F:ATGAGCTACTGCAGGAAGAA R:TTCATGGTTAAAGTTGGGTAA	(CA)n	8	205-223	1.87	0.3147	0.3483	0.2898	This study, based on U74637
test18	F:AGGAGACTGGACCTTAGACC R:AATGGCACTTATCACTTTGG	(CA)n	8	137-151	3.26	0.7133	0.5337	0.6724	This study, based on U74639
test24	F:GTCACAAAAGAGTCGGACAT R:GACAGTGCTGGCCTAGATAC	(TG)n(TA)n	7	143-161	2.86	0.5937	0.4121	0.5633	This study, based on L22193 [30]
test26	F:GGTACCGCAAAGTAATTCAT R:CCATGGAGTCACAAAGAGTT	(CA)n	7	130-142	3.30	0.6971	0.6286	0.6657	This study, based on L22198 [30]
AF5	F:GTGGGAAGAGATAGAGGAAGC R:GAGCCACAAGGCACAGCCAAC	(GT)n	9	158-176	3.45	0.7307	0.6139	0.6984	X82615 [23]
TGLA378	F:TTTATAGCCAACCATATACTTTGCC R:CAGTACCTCTCAACTTCATGTATGT	(CA)n	7	120-134	2.54	0.5218	0.4910	0.486	[23]

N: allele number; AR: allele richness; He: expected heterozygosity; Ho: observed heterozygosity

Table S3. Likelihood of simulations in STRUCTURE 2.3.3 as inferred by STRUCTURE HARVESTER 0.6.93 under different choices of cluster number (K).

K	Reps	Mean LnP(K)	Stdev LnP(K)	Ln'(K)	|Ln''(K)|	Delta K
2	2	-4108.90	19.94	NA	NA	NA
3	2	-4051.00	0.57	57.90	1.15	2.03
4	2	-3994.25	4.17	56.75	36.75	8.81
**5**	**2**	**-3974.25**	**2.33**	**20.00**	**26.10**	**11.19**
6	2	-3980.35	5.44	-6.10	45.50	8.36
7	2	-4031.95	53.53	-51.60	58.00	1.08
8	2	-4025.55	21.14	6.40	15.40	0.73
9	2	-4034.55	21.57	-9.00	28.30	1.31
10	2	-4071.85	37.83	-37.30	NA	NA

Table S4. Assignment probabilities of each *P. przewalskii* patch to each of the clusters estimated in STRUCTURE (K=5) and the corresponding assignment results in GENELAND (K=4).

	**STRUCTURE (K=5)**						**GENELAND**
	PopS1	PopS2	PopS3	PopS4	PopS5		**(K=4)**
B	**0.691***	0.089	0.020	0.107	0.094	PopS1	PopG1
D	0.122	0.176	0.037	**0.395**	0.271	PopS4	PopG2
H	0.171	0.208	0.038	0.172	**0.412**	PopS5	PopG2
J	**0.782**	0.067	0.030	0.038	0.083	PopS1	PopG1
N	0.039	**0.699**	0.017	0.103	0.143	PopS2	PopG2
S	0.082	0.211	0.022	0.177	**0.508**	PopS5	PopG2
T	0.015	**0.854**	0.013	0.051	0.067	PopS2	PopG2
W	0.091	0.071	**0.750**	0.055	0.033	PopS3	PopG3
Y	0.088	0.107	0.032	**0.710**	0.062	PopS4	PopG4

*These numbers indicate percentage of individuals assigned to each group

Table S5. Population pairwise genetic distance estimated by STRUCTURE 2.3.3 based on differences in microsatellite allele frequency.

	**PopS1**	**PopS2**	**PopS3**	**PopS4**
**PopS2**	0.0608	-	-	-
**PopS3**	0.0866	0.0730	-	-
**PopS4**	0.0637	0.0438	0.0657	-
**PopS5**	0.0460	0.0342	0.0886	0.0287

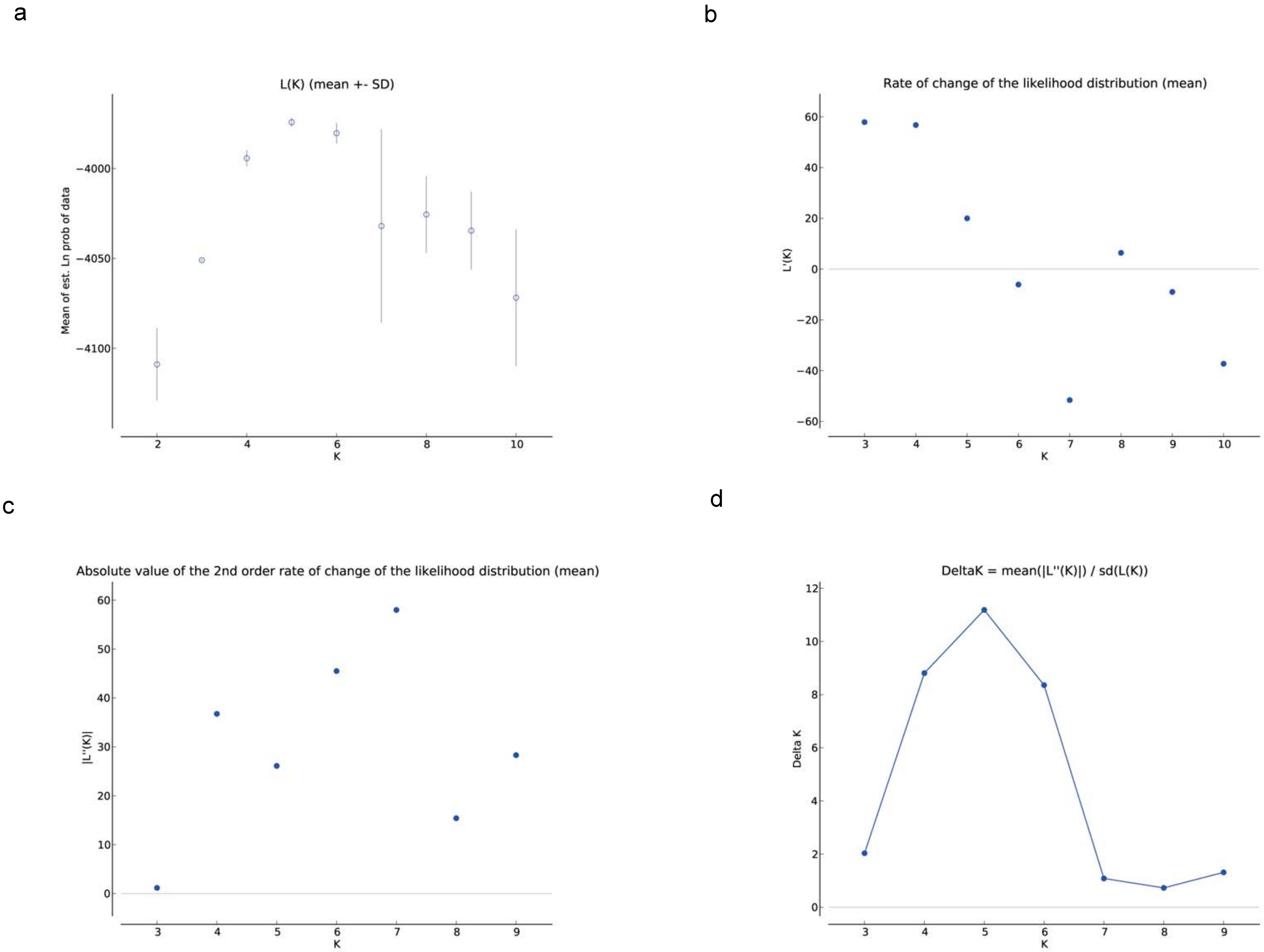
a
L(K) (mean +- SD)
Mean of est. Ln prob of data
−4000
−4050
−4100
2
4
6
8
10
K
b
Rate of change of the likelihood distribution (mean)
L'(K)
60
40
20
0
−20
−40
−60
3
4
5
6
7
8
9
10
K
c
Absolute value of the 2nd order rate of change of the likelihood distribution (mean)
|L''(K)|
60
50
40
30
20
10
0
3
4
5
6
7
8
9
K
d
DeltaK = mean(|L''(K)|) / sd(L(K))
Delta K
12
10
8
6
4
2
0
3
4
5
6
7
8
9
K

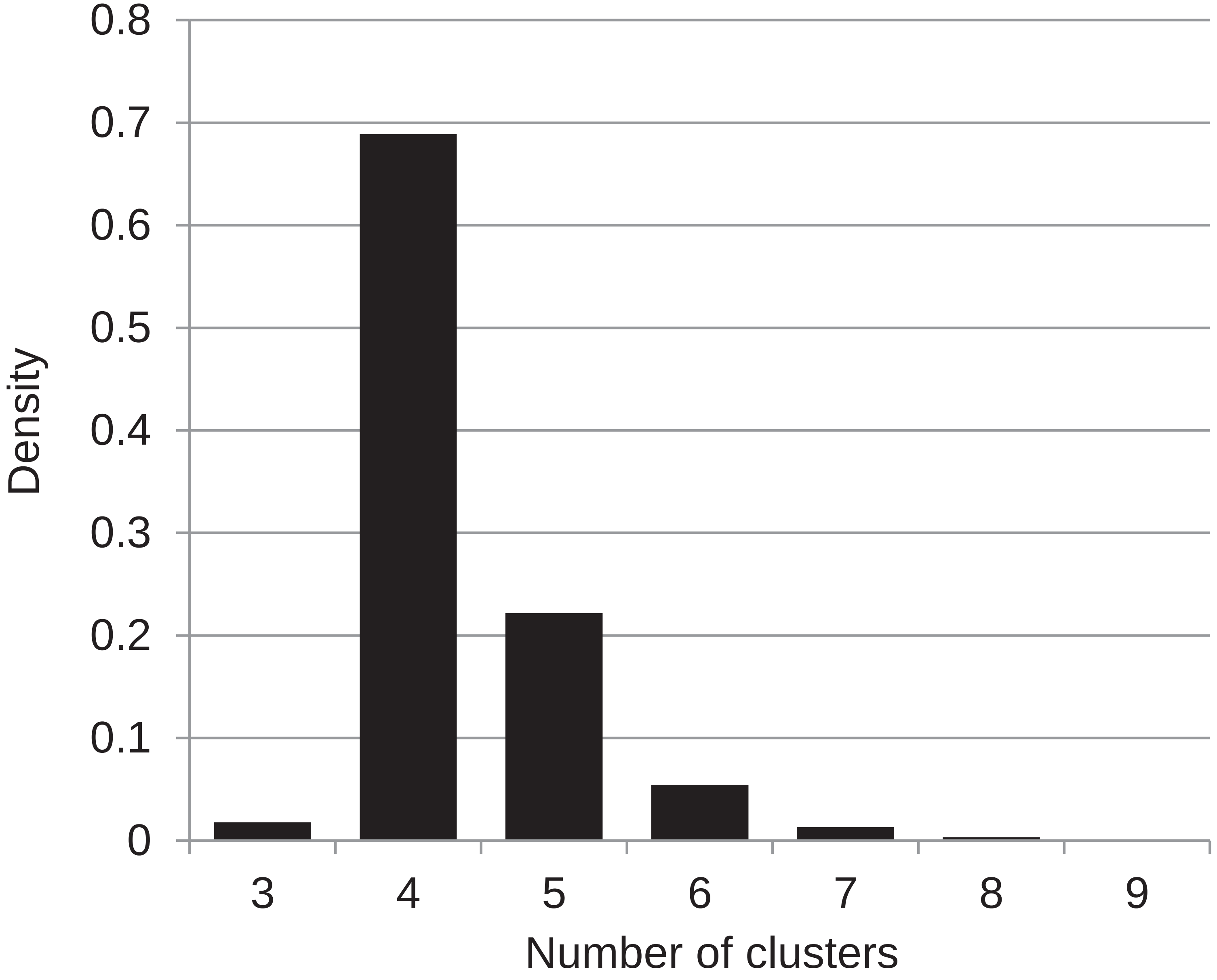
0.8
0.7
0.6
0.5
0.4
0.3
0.2
0.1
0
Density
3
4
5
6
7
8
9
Number of clusters
